# Fine-scale succession patterns and assembly mechanisms of bacterial community of *Litopenaeus vannamei* larvae across the developmental cycle

**DOI:** 10.1186/s40168-020-00879-w

**Published:** 2020-07-03

**Authors:** Yanting Wang, Kai Wang, Lei Huang, Pengsheng Dong, Sipeng Wang, Heping Chen, Zheng Lu, Dandi Hou, Demin Zhang

**Affiliations:** 1 State Key Laboratory for Managing Biotic and Chemical Threats to the Quality and Safety of Agro-products, Ningbo University, Ningbo, 315211 China; 2 School of Marine Sciences, Ningbo University, Ningbo, 315211 China; 3 School of Civil and Environmental Engineering, Ningbo University, Ningbo, 315211 China; 4 Huzhou Southern Taihu Lake Agricultural Biotechnology Institute, Huzhou, 313000 China

**Keywords:** Shrimp larvae, Early life microbiome, Succession pattern, Community assembly, Host development

## Abstract

**Background:**

Microbiome assembly in early life may have a long-term impact on host health. Larval nursery is a crucial period that determines the success in culture of *Litopenaeus vannamei*, the most productive shrimp species in world aquaculture industry. However, the succession patterns and assembly mechanisms of larval shrimp bacterial community still lack characterization at a fine temporal scale. Here, using a high-frequency sampling strategy and 16S rRNA gene amplicon sequencing, we investigated dynamics of larval shrimp bacterial community and its relationship with bacterioplankton in the rearing water across the whole developmental cycle in a realistic aquaculture practice.

**Results:**

Alpha-diversity of larval shrimp bacteria showed a U-shaped pattern across the developmental cycle with the stages *zoea* and *mysis* as the valley. Correspondingly, the compositions of dominant bacterial taxa at the stages *nauplius* and early *postlarvae* were more complex than other stages. Remarkably, *Rhodobacteraceae* maintained the overwhelming dominance after the mouth opening of larvae (*zoea* I~early *postlarvae*). The taxonomic and phylogenetic compositions of larval bacterial community both showed stage-dependent patterns with higher rate of taxonomic turnover, suggesting that taxonomic turnover was mainly driven by temporal switching among closely related taxa (such as *Rhodobacteraceae* taxa). The assembly of larval bacteria was overall governed by neutral processes (dispersal among individuals and ecological drift) at all the stages, but bacterioplankton also had certain contribution during three sub-stages of *zoea*, when larval and water bacterial communities were most associated. Furthermore, the positive host selection for *Rhodobacteraceae* taxa from the rearing water during the *zoea* stage and its persistent dominance and large predicted contribution to metabolic potentials of organic matters at post-mouth opening stages suggest a crucial role of this family in larval microbiome and thus a potential source of probiotic candidates for shrimp larval nursery.

**Conclusions:**

Our results reveal pronounced succession patterns and dynamic assembly processes of larval shrimp bacterial communities during the developmental cycle, highlighting the importance of the mouth opening stage from the perspective of microbial ecology. We also suggest the possibility and potential timing in microbial management of the rearing water for achieving the beneficial larval microbiota in the nursery practice.

Video Abstract

## Background

The microbiota of animals is closely related to their health status [1, 2], nutrient metabolism [3–5], and immune system [6, 7]. After animals’ hatch or birth, their intestinal, skin, and oral microbial communities are gradually assembled. Microbial community assembly in early life may have a long-term impact on host health. Some studies have found that microbiome dysbiosis in infants and young children is associated with obesity [8], inflammatory bowel disease (IBD) [9], and immune diseases [10, 11]. In recent years, the relationship between the intestinal microbial community and the growth or health of aquatic invertebrates (such as shrimp) has been concerned [12–17]. As the most productive shrimp species in world aquaculture industry, the Pacific white shrimp (*Litopenaeus vannamei*) culture is mainly restricted by unstable quality of larvae and frequent outbreak of diseases [18–20]. Larval nursery, covering *nauplius*, *zoea*, *mysis*, and early *postlarvae* stages, is a crucial process that largely determines the success of Pacific white shrimp culture. The quality of larvae is closely related to the growth, development, and resistance to stress/disease of shrimps in subsequent culture stages [21]. At present, the Pacific white shrimp microbiome research is mainly focused on other growth stages (i.e., juvenile, sub-adult, or adult) in terms of their associations with outbreak of disease [15, 16, 22–24], growth [12, 25], and stress-resistance [13, 26]. Furthermore, some studies have reported changes in the structure and function of the intestinal microbial community of postlarvae, juvenile and/or adult shrimp with development [17, 23, 27–30]. The understanding about how the microbiota of Pacific white shrimps functioning in their early life relies on revealing the succession and assembly mechanism of larval microbial community.

Intestinal microbiota of postlarvae, juvenile or adult shrimps could be determined by the developmental stage [17, 23, 27, 28, 30], genetic characteristics [14, 31], health status [17, 22–24], and habitat [13, 32, 33], and are commonly dominated by *α-Proteobacteria*, *γ-Proteobacteria*, *Firmicutes*, *Bacteroidetes* and *Actinobacteria* [13, 23, 28, 33–35]. For example, Xiong et al. found that the relative abundance of *α-Proteobacteria* decreased with the development of *L. vannamei*, but *Actinobacteria* showed an opposite trend [23]. In addition, many studies have found that *Rhodobacteraceae* taxa are ubiquitous in the intestinal microbiota of juvenile or adult shrimps [13, 23, 28, 34]. Some studies further observed the higher relative abundance of *Rhodobacteraceae* in the intestinal tract of healthy [17, 36, 37] and cold-resistant shrimps [13] compared with that of diseased and cold-vulnerable ones, respectively. Intestinal bacterial communities of juvenile or adult shrimps and bacterioplankton in the rearing water often significantly differ [17, 38, 39]. A recent work even demonstrated that very few shrimp intestinal bacteria were derived from the rearing water [38]. However, the bacterial community composition of larval shrimp and its relationship with habitat bacteria are unclear. To the best of our knowledge, only several studies have focused on the bacterial community of *L. vannamei* larvae. For example, Zheng et al. found that *α-Proteobacteria*, *γ-Proteobacteria*, and *Bacteroidetes* species could be widely isolated from larval shrimp samples [40], while based on 16S rRNA gene sequencing, they found larval bacterial communities persistently dominated by *Enterobacteriaceae* across developmental stages (> 85% in relative abundance) [41]. However, another group reported a significant succession pattern in larval shrimp microbiota with host development [42]. These previous studies with relatively low sampling frequency have yielded controversial results, indicating that an investigation at a fine temporal scale into larval shrimp microbiota in aquaculture practice is needed.

Unveiling the assembly mechanism of shrimp microbiota can help resolve the debate on whether we could improve the success rate of shrimp culture via manipulating their microbiota. Microbial community assembly is generally governed by two categories of ecological processes: deterministic and stochastic. Deterministic processes include abiotic selection and biological interaction, while stochastic processes (also known as neutral processes) include dispersal-related processes and ecological drift [43, 44]. Recently, neutral models have been used to disentangle the assembly processes of each species in host microbial communities [17, 43, 45, 46]. This model assumes that individuals in a local community could be randomly lost and then replaced by other members in the community and/or supplemented from metacommunity (species pool) via dispersal [46, 47]. The neutral model infers assembly processes by fitting the relationship between the occurrence frequency of species in the local communities and their abundance in the metacommunity [46, 48]. The species with occurrence frequency deviated from that predicted by the model are considered to be selected for or against by the local community, or have a different ability to disperse compared with the neutrally distributed species [43, 46]. Using this approach, Burns et al. found that the contribution of neutral processes to the assembly of zebrafish intestinal bacteria declined with host development [46], while the importance of neutral processes in shaping intestinal bacterial communities increases with the age of shrimp (from postlarvae to adult) in culture practice, but declined with disease outbreak [17]. However, little is known about the dynamics and taxonomic dependency of assembly processes of bacteria in larval shrimp.

In this study, we used 16S rRNA gene amplicon sequencing to investigate the succession and assembly processes of *L. vannamei* larval bacterial community in a realistic aquaculture practice with sufficient biological replicates. A high-frequency sampling strategy was applied to collect shrimp larvae (from the fertilized eggs of a pair of parents) and rearing water samples across *nauplius*, *zoea* I, *zoea* II, *zoea* III, *mysis*, and early *postlarvae* stages lasting for 15 days (The sampling details are shown in Additional file 2: Figure S1). Using multivariate analyses, the neutral model, and functional prediction (with PICRUSt2 [Phylogenetic Investigation of Communities by Reconstruction of Observed States] [49]), we aimed to reveal the following: (1) the dynamics of α-diversity, composition, and predicted functional potentials of larval shrimp bacterial community with host development, (2) the taxonomic and phylogenetic succession pattern of larval bacterial community, (3) the dynamics and taxonomic dependency in assembly processes of larval shrimp bacteria across developmental stages, and (4) to what extent the rearing water bacterioplankton can influence the assembly of larval shrimp bacteria.

## Results

### Alpha-diversity of bacterial community

**Fig. 1:**
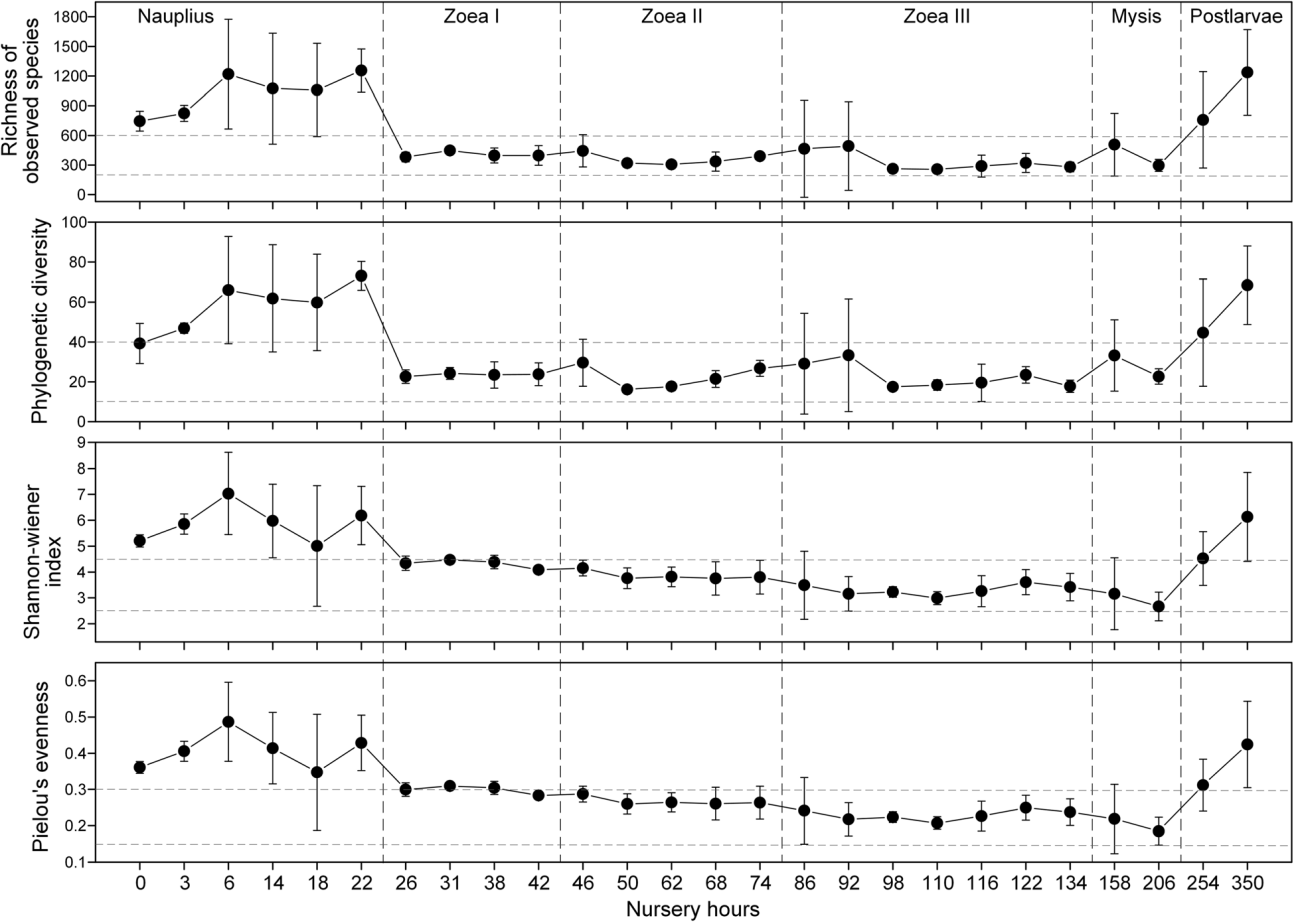
Fine-scale temporal dynamics of α-diversity and evenness indices of larval shrimp bacterial communities. Data present means ± standard deviation

The bacterial α-diversity indices of shrimp larvae and rearing water showed dramatic variability with host development. Bacterial α-diversity and evenness of larvae were at a high level at *nauplius* stage, then decreased at *zoea* I stage, and finally reverted to the initial level at early *postlarvae* stage (Fig. 1, Additional file 2: Figure S2). Species richness and phylogenetic diversity decreased sharply from *nauplius* to *zoea* I (*P* < 0.05), and then remained stable until *mysis* stage, while Shannon and Pielou’s evenness indices showed a declining trend from *nauplius* to *mysis* (Additional file 2: Figure S2). At the finer time-scale, bacterial α-diversity showed certain fluctuation even in a short period of time, especially during the stages *nauplius* and early *postlarvae* (Fig. 1).

Species richness and phylogenetic diversity of bacterioplankton in the rearing water showed somewhat increasing trend with host development, but relatively stable and significantly lower compared with those of larvae at the stages *nauplius*, *zoea* I–II, and early *postlarvae* (Additional file 2: Figure S2). The Shannon and evenness indices of larval bacterial community were significantly higher than that of bacterioplankton at the stages *nauplius* and *zoea* I–II, but showed an opposite pattern at the stages *zoea* III and *mysis*. Finally, α-diversity of bacterioplankton reached the highest level at early *postlarvae* stage.

### Dynamics of dominant bacterial taxa in shrimp larvae

**Fig. 2:**
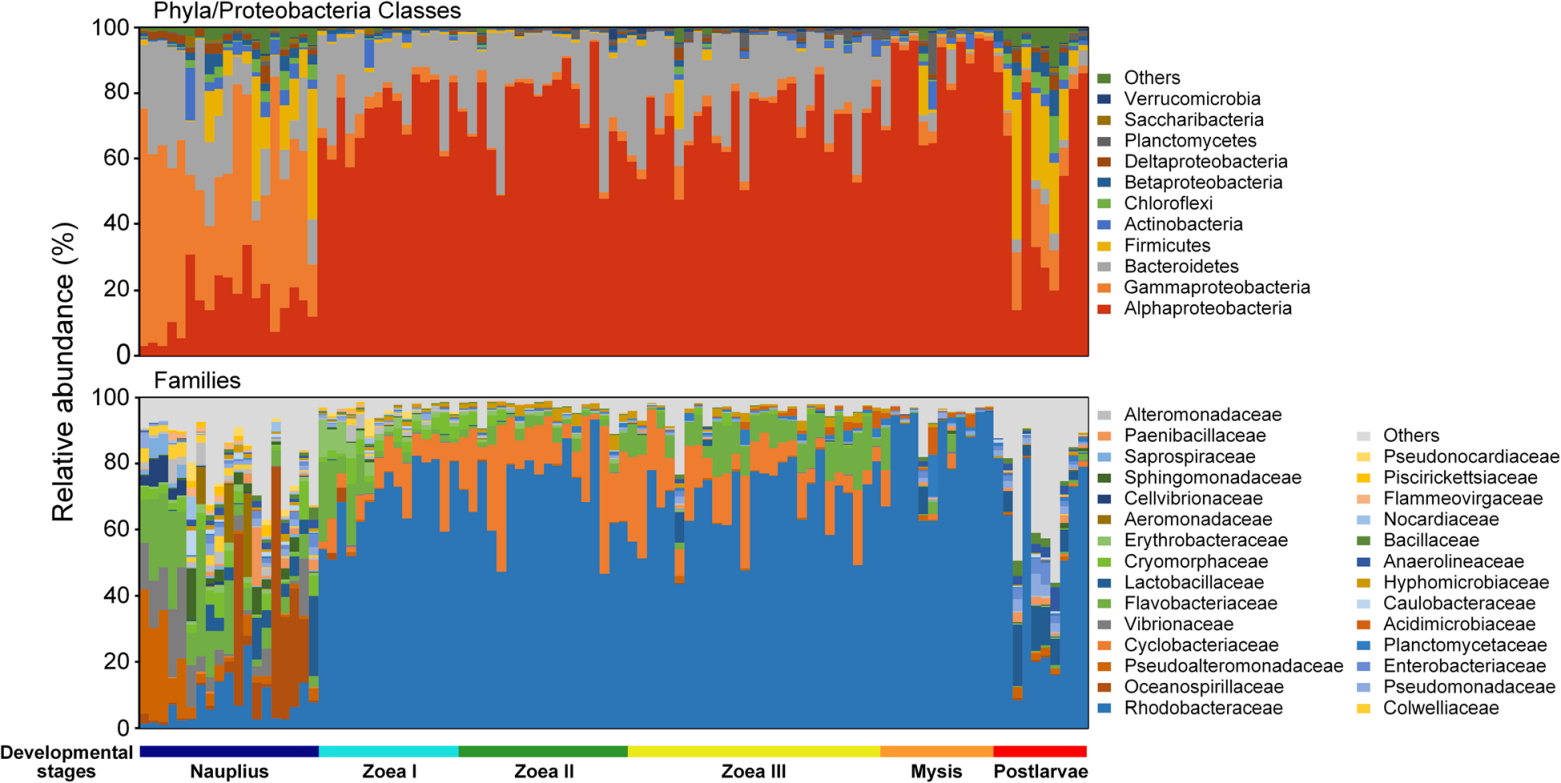
Dynamics of dominant phyla/proteobacterial classes and families of larval shrimp bacterial communities. The phyla and proteobacterial classes with average relative abundant > 1% and the families with average relative abundant > 2% at least in one sampling time point are shown

The bacterial communities of naupliar shrimps mainly dominated by *γ-Proteobacteria* (43.6% in average relative abundance), *Bacteroidetes* (20.0%), *α-Proteobacteria* (15.7%), and *Firmicutes* (8.5%) (Fig. 2). At *zoea* stage, the dominant groups shifted to *α-Proteobacteria* (72.4%, mainly *Rhodobacteraceae*, 69.8%) and *Bacteroidetes* (20.8%, including *Cyclobacteriaceae*, 12.8%), while the relative abundance of *α-Proteobacteria* further increased to the extreme dominance (86.2%, mainly *Rhodobacteraceae*, 84.9%) at *mysis* stage. However, the average relative abundance of *Rhodobacteraceae* decreased to 58.3% at early *postlarvae* stage, while *Firmicutes* (15.8%), *γ-Proteobacteria* (9.9%), and *Chloroflexi* (3.6%) were enriched. Overall, the composition of dominant bacterial groups (at the phylum or family level) at the stages *nauplius* and *postlarvae* were more complex than other stages. Linear discriminant analysis effect size (LEfSe) showed the discriminatory taxa of larval shrimp microbiota at different stages, with the class *Sphingobacteria* (mainly the family *Saprospiraceae*), the class *Flavobacteria* (including the genera *Pseudofulvibacter*, *Nonlabens*, and *Crocinitomix*), *Actinobacteria* (including the genera *Pseudonocardia* and *Gordonia*), and *γ-Proteobacteria* (including the genera *Vibrio*, *Reinekea*, and *Pseudoalteromonas*) more abundant at *nauplius* stage; the class *Cytophagia* (including the genus *Algoriphagus*) and two *Rhodobacteraceae* genera *Roseovarius* and *Donghicola* at *zoea* stage; the genus *Ilumatobacter*, the family *Rhodobacteraceae*, and *Planctomycetes* (including the genus *Rhodopirellula*) at *mysis* stage; *Firmicutes* (including the order *Clostridiales* and the genera *Lactobacillus* and *Bacillus*), the genus *Pseudomonas*, the family *Enterobacteriaceae*, and a *Rhodobacteraceae* genus *Ruegeria* at early *postlarvae* stage (Additional file 2: Figure S3).

**Fig. 3:**
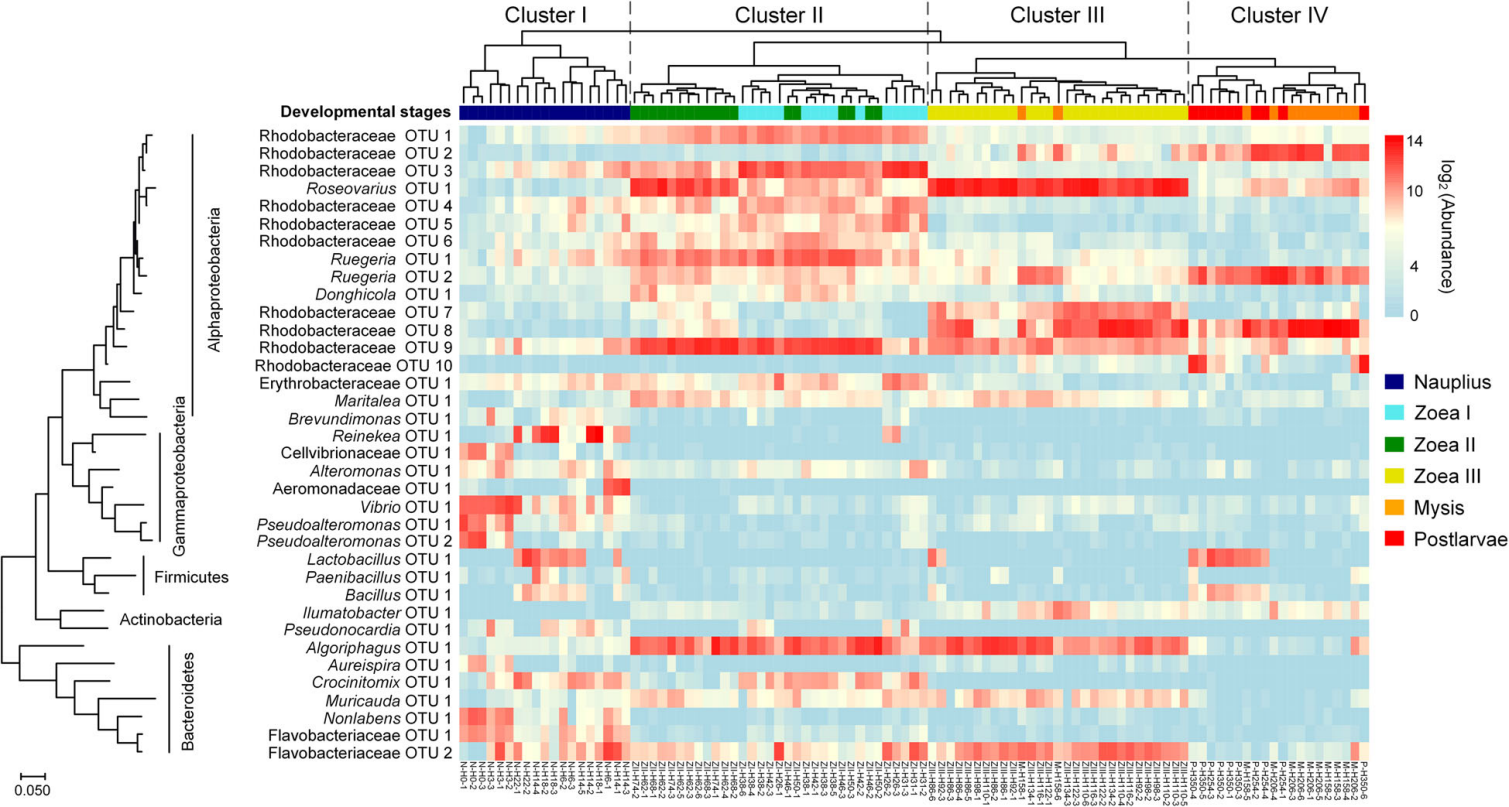
Heatmap showing the dynamics of dominant larval bacterial OTUs across developmental stages. The OTUs with average abundance > 2% at least in one sampling time point are shown. The data of OTU abundance was log_2_ transformed. The phylogenetic tree was built using the maximum likelihood method in MEGA 7.0

The heatmap shows the succession pattern of dominant bacterial OTUs (operational taxonomic units) of shrimp larvae, and 89.1% of samples could be classified into four clusters according to the developmental stage: cluster I (*nauplius*), cluster II (*zoea* I and II), cluster III (*zoea* III), cluster IV (*mysis* and *postlarvae*) (Fig. 3). It is worth noting that the composition of dominant OTUs were complex before the mouth opening of larvae. However, after the mouth opening, the composition of dominant OTUs tended to be simple, when the OTU turnover was largely represented by switching among *Rhodobacteraceae* taxa. Specifically, 10 *Rhodobacteraceae* OTUs were predominated in cluster II, but only 2 of them remained predominant in cluster III with other two emerging ones, and then a subset of dominant *Rhodobacteraceae* OTUs in the 2 previous stages were present in cluster IV.

### Taxonomic and phylogenetic turnover of bacterial community with host development

**Fig. 4:**
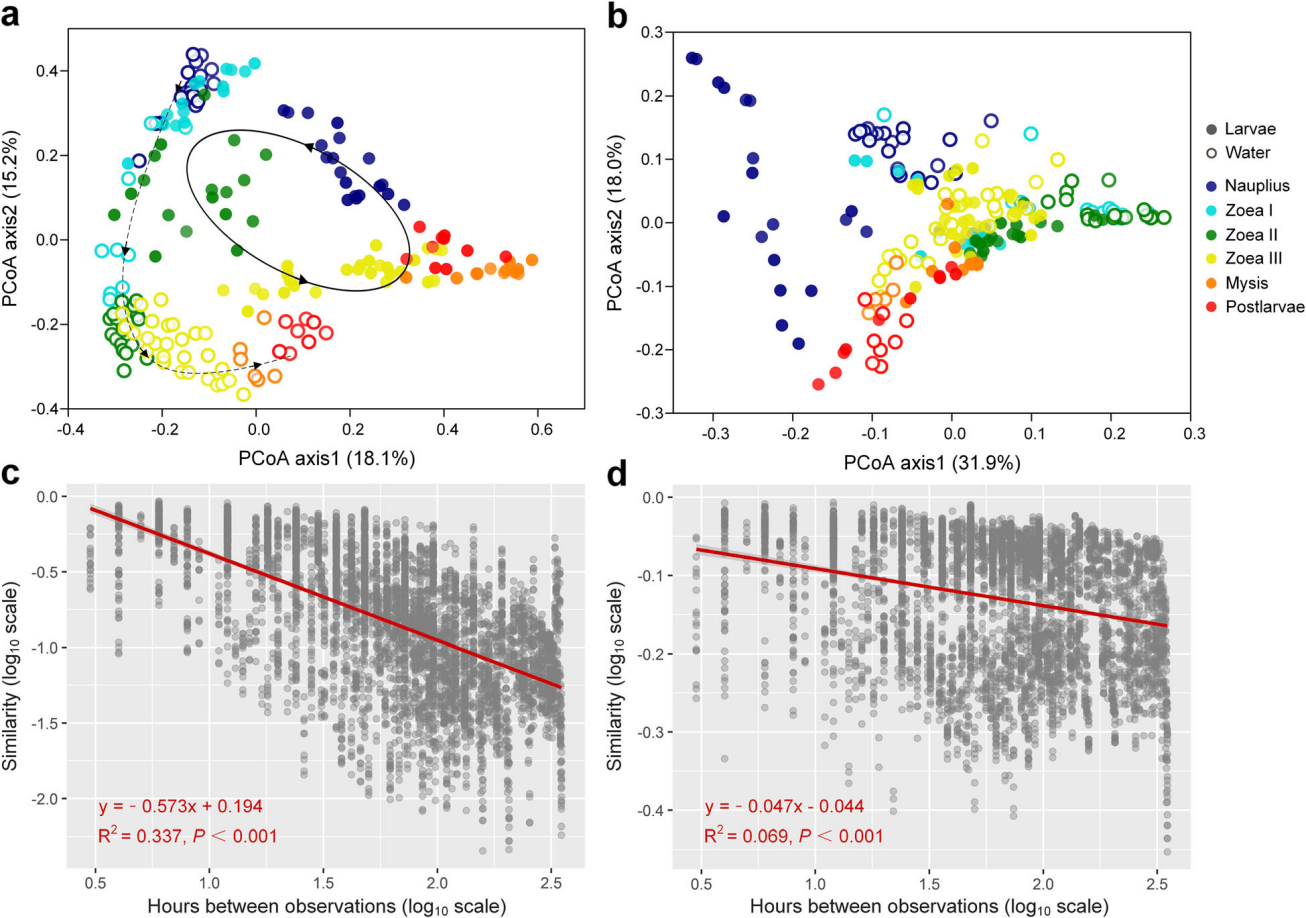
Taxonomic and phylogenetic turnover of larval and water bacterial communities with host development. Principal coordinate analysis (PCoA) visualizing compositional variations of larval and water bacterial communities across developmental stages based on Bray-Curtis dissimilarity (**a**) and weighted UniFrac distance (**b**). Time-decay in similarity between larval bacterial communities based on Bray-Curtis similarity (**c**) and phylogenetic similarity based on UniFrac distance (1 - weighted UniFrac distance) (**d**). The data of similarity values and hours between observations were shown as log_10_ transformed

In general, bacterial community compositions of shrimp larvae and rearing water were both clustered according to developmental stages (Fig. 4, Additional file 1: Table S1). The taxonomic composition of bacterial community in larvae and water showed distinct successional trajectories, while the phylogenetic turnover trajectories of two communities overlapped to some extent during the sub-stages of *zoea* (Fig. 4a, b). As evidenced by One-way analysis of similarity (ANOSIM), the compositions of larval and water bacterial communities were significantly different at any stages (all *P* < 0.01, Additional file 1: Table S2). Furthermore, the succession of larval bacterial community taxonomically and phylogenetically fitted the time-decay model (all *P* < 0.001) with a much greater rate of OTU turnover (*w* = − 0.573, *R*^2^ = 0.337) compared with phylogenetic turnover rate (*w* = − 0.047, *R*^2^ = 0.069) (Fig. 4c, d).

### The relationship between larval and water bacterial communities

We observed overall low taxonomic similarity but high phylogenetic similarity between larval and water bacterial communities (Additional file 2: Figure S4). Before the mouth opening (*nauplius*), the similarity between larval and water communities was low (19.0% in average taxonomic similarity and 65.0% in phylogenetic similarity), and rose to the highest level (45.2% and 83.1%) after the mouth opening (*zoea* I). But it decreased to the initial level at later stages (*mysis* and early *postlarvae*). In addition, we found that larval bacterial communities at the stages *zoea* I-II and *mysis* showed higher similarities and/or more shared OTUs with the water bacterial communities from the previous stage compared with that at the same stage (*P* < 0.01, Additional file 2: Figure S5).

### Fit of the neutral model for larval shrimp bacteria

**Fig. 5:**
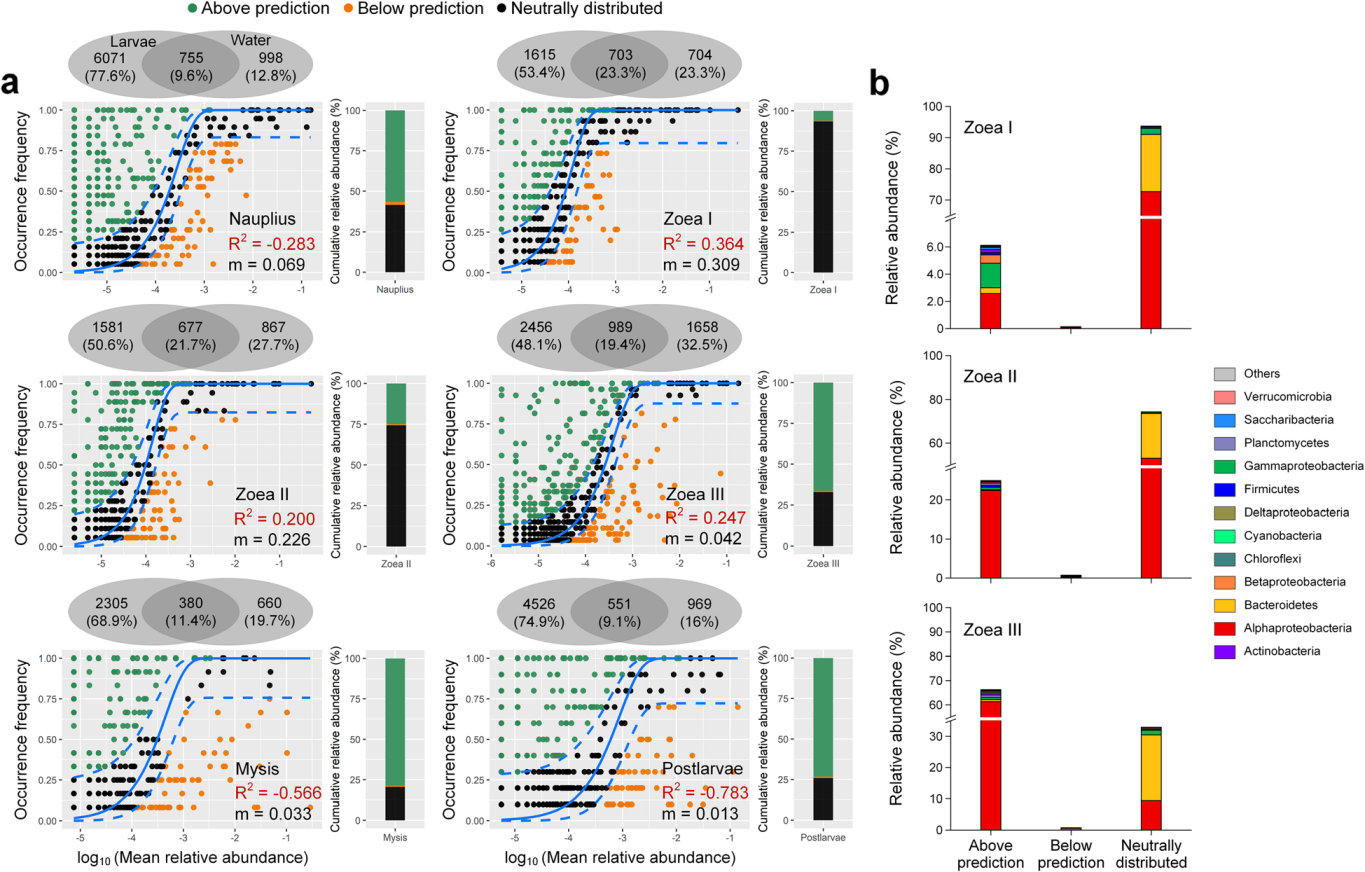
Fit of the neutral models for larval bacterial communities with corresponding bacterioplankton as the source. **a** The Venn diagrams show the number and proportion of OTUs being unique in shrimp larvae/rearing water and shared by larvae and water. The OTUs that occurred more frequently than predicted by the model are shown in green, while those occurred less frequently than predicted are shown in orange. Blue dashed lines represent 95% confidence intervals around the model prediction and the OTUs fall within the confidence intervals were considered as neutrally distributed. *R*^2^ values present the goodness of fit of the neutral model, ranging from 0 (no fit) to 1 (perfect fit). The histograms show the cumulative relative abundance of three categories of OTUs (above prediction, below prediction, and neutrally distributed) in larval bacterial communities at each stage. **b** The taxonomic distribution of three categories of OTUs at three sub-stages of *zoea*, when the occurrence of the larvae-water shared OTUs in larval bacterial communities fitted the model

Before the mouth opening (*nauplius*) and at the later stages (*mysis* and early *postlarvae*), the OTUs shared by larval and water bacterial communities accounted for only 9.1~11.4% of the total OTUs of two communities, but after the mouth opening, the proportion of shared OTUs reached to 19.4~23.3% during *zoea* sub-stages (Fig. 5a). When assuming water bacteria as the source community, the occurrence of the shared OTUs in larval bacterial communities only fitted the neutral model at three sub-stages of *zoea* (*R*^2^ = 0.200~0.364), indicating that bacterioplankton served as one of the important sources of larval bacterial communities during the *zoea* stage. In this period, the cumulative relative abundance of the neutrally distributed OTUs (*zoea* I 93.4%, *zoea* II 74.3%, *zoea* III 33.0%) and the estimated migration rate (*m, zoea* I 0.309, *zoea* II 0.226, *zoea* III 0.042) both gradually decreased (Fig. 5a), and the taxonomic distribution of the three categories OTUs in the neutral model also varied with host development (Fig. 5b). The neutrally distributed OTUs were predominated by *α-Proteobacteria* (mainly *Rhodobacteraceae*) and *Bacteroidetes*, and the relative abundance of *α-Proteobacteria* declined with host development (from 70.2 to 9.3%). The OTUs above prediction were mainly from *α-Proteobacteria* (mainly *Rhodobacteraceae*) and *γ-Proteobacteria*, and the relative abundance of *α-Proteobacteria* dramatically increased from 2.5% to 60.5% with host development.

**Fig. 6:**
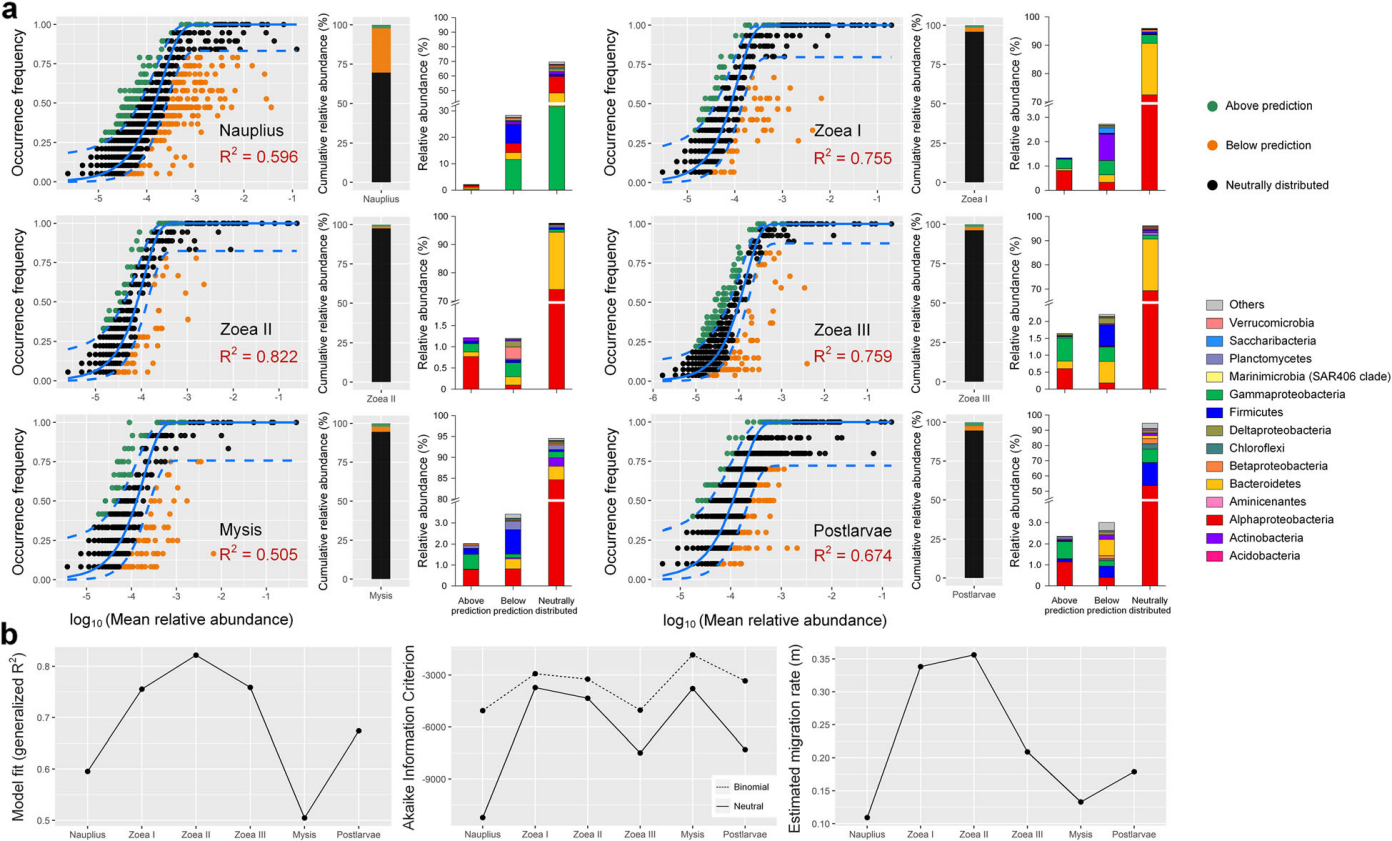
Fit of the neutral models for larval bacterial communities with larval matecommunity as the source. **a** The OTUs that occurred more frequently than predicted by the model are shown in green, while those occurred less frequently than predicted are shown in orange. Blue dashed lines represent 95% confidence intervals around the model prediction and the OTUs fall within the confidence intervals were considered as neutrally distributed. *R*^2^ values present the goodness of fit of the neutral model, ranging from 0 (no fit) to 1 (perfect fit). The cumulative relative abundance and taxonomic distribution of three categories of OTUs (above prediction, below prediction, neutrally distributed) in larval bacterial communities at each stage are shown. **b** The dynamics of *R*^2^ value of the neutral model, Akaike Information Criterion comparing the fit of the neutral model and the binomial distribution model, and the estimated migration rate (*m*) of larval bacterial communities across developmental stages

When assuming larval bacterial metacommunity as the source community, the goodness of fit of the neutral model was largely improved (*R*^2^ = 0.505~0.822) compared with that when assuming bacterioplankton as the source across all stages (Fig. 6), suggesting that exchange of bacteria among larval individuals was a more important source of larval bacterial communities. The cumulative relative abundance and taxonomic distribution of three categories of OTUs in the neutral model varied with host development, especially between pre- and post-mouth opening stages (Fig. 6a). The relative abundance of the neutrally distributed OTUs was 69.6% before the mouth opening (*nauplius*), and sharply increased to 95.9% afterwards (*zoea* I) with more than 94% of OTUs neutrally distributed at each stage. The neutrally distributed OTUs were dominated by *γ-Proteobacteria* (31.5% in average relative abundance), *Bacteroidetes* (16.9%), and *α-Proteobacteria* (11.4%) at *nauplius* stage. After the mouth opening, the neutrally distributed γ-proteobacterial OTUs showed a rapid decrease in relative abundance (*zoea* I 3.0%, *zoea* II 1.0%, *zoea* III 1.5%) and were soon replaced by α-proteobacterial OTUs (*zoea* I 72.5%, *zoea* II 74.1%, *zoea* III 69.3%). The relative abundance of neutrally distributed α-proteobacterial OTUs further increased to 84.6% at *mysis* stage, followed by a decrease in *α-Proteobacteria* (53.7%) and an increase in *Firmicutes* (15.2%) at early *postlarvae* stage. The cumulative relative abundance of the OTUs above prediction was overall low (< 2.4%) across developmental stages, with little changes in the taxonomic distribution. The cumulative relative abundance of the OTUs below prediction before the mouth opening was 28.3%, but dramatically decreased to 3.5% afterwards. At any stages, the assembly of larval bacteria was dominantly governed by neutral processes, and the neutral model performed better than the binomial distribution model (according to Akaike Information Criterion, AIC) (Fig. 6b), suggesting that, except dispersal, ecological drift and dispersal limitation also contributed. In addition, the estimated migration rate (*m*) peaked at the stages *zoea* I–II, and then decreased at the stages *mysis* and *postlarvae* (Fig. 6b), suggesting enhanced dispersal of bacteria among larval individuals right after mouth opening and a stronger dispersal limitation in the later stages.

### Predicted functional profiles of larval shrimp bacterial community

Large differences in functional profiles of larval shrimp bacterial community between pre- and post-mouth opening stages was predicted by PICRUSt2 (Additional file 2: Figure S6a). In general, functional potentials relevant with genetic information processing were enriched in naupliar shrimps compared with larvae at post-mouth opening stages, while many metabolism-relevant potentials (such as biosynthesis of other secondary metabolites and the metabolism of amino acids; carbohydrate; lipid; cofactors and vitamins; and terpenoids and polyketides) were enriched in larvae after the mouth opening. However, some metabolism-relevant potentials somewhat showed a decreasing trend at early *postlarvae* stage. As the predominant bacterial group at the stages *zoea* and *mysis*, the family *Rhodobacteraceae* was predicted to be the major contributor to functional potentials (including metabolism) of larval shrimp bacterial community (Additional file 2: Figure S6b).

## Discussion

### The U-shaped pattern in larval bacterial α-diversity with host development

The α-diversity of larval shrimp bacteria varied with host development, corresponding to previous reports about larvae of aquatic animals such as cod (*Gadus morhua*) [50], gibel carp (*Carassius auratus gibelio*) [51], southern catfish (*Silurus meridionalis*) [52], and zebrafish (*Danio rerio*) [53]. In this study, larval bacterial α-diversity indices all showed a U-shaped pattern with host development. As the larvae are too small to obtain their intestines, the larval microbiota should be mainly derived from the intestinal tract and the fraction attached to the epidermis. At *nauplius* stage, with the release of yolk nutrients and the enlargement of epidermis area, the larval bacterial community could be mainly originated from fertilized eggs (the inheritance of the parents and initial hatching environment) and the epidermis attachment, thus maintaining at a relatively high diversity. When the larvae started eating at *zoea* I stage, their intestinal microbiota began to form, while the larvae molted, imposing the reassembly of larval bacterial community. These changes could lead to the dominance of intestinal bacteria in larval microbiota and thus decrease α-diversity. The α-diversity of larval bacteria was relatively stable across three sub-stages of *zoea* (with a slight declining trend in Shannon index). During this period, the host development (especially the intestinal tract) was largely immature, resulting in low niche diversity that can be provided [51, 54]. This could be a key explanation for the low α-diversity of larval bacteria at that time. In addition, the species richness and phylogenetic diversity of the zoeal bacterial community were very close to that of bacterioplankton in rearing water (Additional file 2: Figure S2). A previous study found that the intestinal microbiota of coho salmon (*Oncorhynchus kisutch*) after first feeding was mainly from water and egg epidermis [55]. We also found that water was an important source of larval bacteria at *zoea* sub-stages (see the Discussion below). At early *postlarvae* stage, α-diversity of larval bacteria bounced back to a similar level at *nauplius* stage, likely due to the more diverse niches provided by the intestinal tract tending to be mature and complex [51, 54].

Shrimp larvae (especially at *zoea* sub-stages) often have weak immune systems and are vulnerable to *zoea-*II syndrome, which can lead to high mortality [56, 57]. Some studies have shown that the diversity of animal intestinal microbiota is closely related to their functional integrity and stability [58, 59]. Although a point of view has suggested that the higher microbial diversity does not necessarily correspond to a more stable and healthy ecosystem [60], high diversity is often considered to hold capability of maintaining the stability and ecological function of microbial community, thus being an important indicator of host health status [61, 62]. A previous work has found that the intestinal bacterial α-diversity of *L. vannamei* with normal growth rates was higher than that of retarded or overgrown shrimps [12]. Moreover, the higher bacterial α-diversity was also observed in the intestinal tract of healthy individuals and the cold-resistant strain of *L. vannamei* relative to that of diseased individuals and the cold-vulnerable strain, respectively [13, 16, 17, 63]. These studies suggest that high bacterial diversity could be a positive signal for maintaining the growth, health, and stress-resistance of shrimps. To some extent, the valley of larval bacterial α-diversity during the *zoea* stage confirms a common empirical view in aquaculture industry that the zoeal shrimps were most vulnerable to the stress and disease in the complete larval developmental cycle [56, 57] on a perspective of microbial ecology, further suggesting that *zoea* is a key stage for ensuring the success of larval nursery.

### Larval bacterial community composition varied with host development

The high-frequency sampling strategy facilitated the unveiling of highly dynamic pattern of larval bacterial communities. The taxonomic and phylogenetic compositions of larval bacteria both showed stage-dependent patterns, even between the sub-stages of *zoea*. This is consistent with the pattern observed in the intestinal bacterial community of fish larvae [50, 52, 53]. In addition, we found distinct compositions and successional trajectories between larval and water bacterial communities. Similar results were often reported in juvenile and adult shrimps (*L. vannamei* and *Macrobrachium nipponense*) [17, 23, 28, 29, 38, 39]. The shrimp larvae with immature digestive system could partially rely on the assistance of bacteria for food digestion and nutrient metabolisms [64], which is corresponding to the enriched metabolic potentials of multiple organic matters in larval shrimp microbiota after the mouth opening (especially at the stages *zoea* and *mysis*), as predicted by PICRUSt2. Thus, the high variability of bacterial community composition might be due to the host’s recruitment of different functional groups for physiological needs [65]. As the morphological and physiological properties of intestinal tract change with host development, the initial “winners” will be reorganized to form a stage-specific bacterial community [66].

Zheng et al. found that *Enterobacteriaceae* were persistently predominant (> 85% in relative abundance) in larval bacterial communities of *L. vannamei* across developmental stages, though *Rhodobacteraceae* were ubiquitous at all stages (second abundant in many of them) [41]. These findings are contrasting to our result, that is, *Enterobacteriaceae* kept at low relative abundance (0.03~2.7%), while *Rhodobacteraceae* maintained the overwhelming dominance after the mouth opening. Unlike the simple composition of dominant bacterial families in Zheng et al. [41], we found more dynamic changes in bacterial community as represented by dramatic fluctuation in relative abundance of *Rhodobacteraceae*, *Cyclobacteriaceae*, and *Flavobacteriaceae* (Fig. 2). Xue et al. have suggested that the choice of DNA extraction kits may result in DNA recovery biases, thus influencing the characterization of larval bacterial community of *L. vannamei* [42]. However, they found *α-Proteobacteria* (*Rhodobacteraceae*), *γ-Proteobacteria*, *Bacteroidetes* (*Flavobacteriaceae*), and *Firmicutes* as the main dominant groups of larval bacterial communities and pronounced succession in community composition, regardless of DNA extraction kits. This fits the general view of our work. Furthermore, they also found the dominance of *Rhodobacteraceae* when using the Stool DNA Kit (Omega, USA), but showing an opposite dynamic pattern (> 80% in relative abundance at *nauplius* stage, followed by a decrease to < 30% in the middle and late stages) compare with that in our study. This suggests that the differences in environmental conditions of nursery systems may also lead to distinct patterns in larval microbiota dynamics. Future efforts are needed to reveal the impact of local environmental conditions on the succession of larval shrimp bacterial communities.

Many studies have found that host development [30, 53, 67] and diet [35, 51] largely shape the intestinal microbiome of aquatic animals. In this study, the shifts of physiological state, nutritional intake mode, and microbial source between pre- and post-mouth opening stages likely led to dramatic differences in larval bacterial communities. It is worth noting that, after the mouth opening, the OTU composition of bacterial community rapidly varied, but the turnover of dominant OTUs mainly occurred within the family *Rhodobacteraceae* (Fig. 3). Furthermore, the time-decay model showed that the OTU turnover rate of larval bacterial community was much higher than phylogenetic turnover rate. These results suggest that the taxonomic turnover of larval bacteria was mainly driven by temporal switching among closely related taxa. Many studies have found *Rhodobacteraceae* persistently dominant in the intestinal tract of *L. vannamei* across juvenile to adult stages (ranging from 6 to 50% in average relative abundance) [13, 17, 23, 28, 34]. Therefore, the family *Rhodobacteraceae* is likely a core group of intestinal microbiota of *L*. *vannamei*. *Rhodobacteraceae* are heterotrophic bacteria with extremely high diversity and versatility in organic matter degradation, and widely distributed across various marine ecosystems [68–70]. Most *Rhodobacteraceae* taxa are able to synthesize vitamin B_12_, which is a dietary essential for shrimps [71]. Moreover, functional prediction showed that the family *Rhodobacteraceae* largely contributed to the potentials in biosynthesis and the metabolism of multiple organic matters after the mouth opening of larvae, indicating that they may participate in the metabolism of organic matters in the digestive tract of larvae and/or provide essential nutrients for host growth. The relative abundance of *Rhodobacteraceae* in the intestinal bacterial community of healthy *L. vannamei* individuals is often higher than that of diseased ones, and shows an antagonistic relationship with potential pathogens such as *Vibrio* [36, 72, 73]. One proven example is that strains of the species *Ruegeria* sp. and *Phaeobacter* sp. (both affiliated to *Rhodobacteraceae*) can produce tropodithietic acid (TDA) to inhibit *Vibrio anguillarum*, thus holding probiotic potential [74–76]. In addition, Xiong et al. found that the relative abundance of *Rhodobacteraceae* in the intestinal bacterial community of *L. vannamei* individuals with normal growth rate was higher than that of retarded or overgrown ones [12]. The higher relative abundance of *Rhodobacteraceae* was also observed in the intestinal tract of cold-resistant strain of *L. vannamei* relative to cold-vulnerable strain [13]. Collectively, we speculate that the dramatic enrichment of *Rhodobacteraceae* (including some *Ruegeria* taxa) after the mouth opening of larvae may play a positive role in promoting digestion, providing nutrients, and inhibiting pathogens. Furthermore, the temporal switching among *Rhodobacteraceae* taxa suggests distinct assemblages of *Rhodobacteraceae* taxa could be recruited for maintaining certain functions such as the metabolism of different organic matters derived from the partially modified feeds at different stages. Thus, the family *Rhodobacteraceae* might be a potential source of probiotics for larval shrimp nursery. To test these hypotheses, using metagenomics and metatranscriptomics to study the metabolic potentials of distinct *Rhodobacteraceae* taxa and how they functioning at various developmental stages of shrimp larvae should be considered as an important future direction.

### Neutral processes dominated the assembly of larval bacteria

According to the fitting of the neutral model, we found that larval bacterial communities mainly sourced from the larval metacommunity, while water bacterioplankton community only had certain contribution at *zoea* sub-stages. In general, the neutral processes dominantly governed the assembly of larval bacteria, corresponding to the findings in zebrafish larvae [53]. These results suggest that the assembly of larval bacteria overall depends on the exchanges among individuals, probably via cross-feeding of feces and/or bioflocs [24, 77, 78]. The influence of host morphology on the assembly processes of microbiota has been demonstrated in many kinds of fishes [53, 65]. When larval shrimps are undergoing continuous metamorphic development with frequent molting and feed replacement, their bacterial communities are also undergoing frequent reassembly. Coupled with their physiological immaturity, the relative abundance of OTUs above the neutral prediction was low, suggesting the overall weak host selection. It is worth noting that the relative abundance of OTUs below the prediction was the highest before the mouth opening (*nauplius*), when the migration rate (*m*) was the lowest (Fig. 6). This suggests that dispersal limitation was strong at this stage, because bacteria could not be exchanged among larval individual via feeding. The substantial improvement of the neutral model’s fitting goodness over the binomial distribution model at this stage further confirmed the importance of dispersal limitation. After the mouth opening, especially at the stages *zoea* I–II, the fitness of the neutral model (*R*^2^) rapidly increased, the migration rate rose to the highest level, and the gap of fitness between the two models decreased, suggesting that dispersal process dominated the assembly of larval bacteria. In the subsequent stages, the migration rate and relative abundance of neutrally distributed OTUs showed a declining trend, probably because the improvement in matureness of shrimp larvae led to the enhancement of host selection on microbiota. In addition, the compositions of OTUs neutrally distributed or deviated from neutral prediction between pre- and post-mouth opening stages were dramatically different. These results reveal the remarkable succession pattern and the dynamics in assembly processes of larval bacterial communities, emphasizing the importance of the mouth opening stage of larval shrimp from the ecological perspective.

Many studies have found that the initial establishment of host microbiome can be affected by the surrounding environment [38, 79, 80]. We also found rearing water as a source of larval bacterial community at three sub-stages of *zoea*, which can be considered as the beginning of establishment of larval intestinal microbiota (Fig. 5). At the *zoea* stage, the proportion of shared OTUs and composition similarity between larval and water bacterial communities reached the highest level, as was the estimated migration rate of water bacteria to the larval community, suggesting that larval and water bacterial communities were most associated in this period. Therefore, *zoea* may be a critical stage for the regulation of larval microbiota through manipulating the microbial community of rearing water. On the other hand, it is particularly important to ensure the microbial safety of rearing water (such as prevention of pathogenic bacteria) after the mouth opening of larvae. In addition, compared with the water bacterial community at the same stage, the larval bacterial communities of *zoea* I–II and *mysis* all showed a stronger association with the water community from the previous stage (Additional file 2: Figure S5). Similar time lag in colonization of environmental bacteria into zebrafish has been observed [53]. This suggests that the regulation of larval microbiota by microbial management of rearing water in aquaculture practice should be launched before the mouth opening of shrimp larvae.

Taken bacterioplankton as the source for the neutral model fitting, the relative abundance of the neutrally distributed OTUs and the migration rate gradually decreased during *zoea* sub-stages, indicating that the dominant process governing the colonization of bacterioplankton into larval communities shifted from dispersal to host selection. The relative abundance of OTUs above prediction gradually increased during *zoea* sub-stages (*zoea* I 6.1%; *zoea* II 24.9%; *zoea* III 66.3%) (Fig. 5), especially *Rhodobacteraceae* OTUs (*zoea* I 2.0%, *zoea* II 22.3%, *zoea* III 61.1%), suggesting that *Rhodobacteraceae* taxa in the rearing water can adapt to the internal environment of larvae, and be positively selected by the host. Knowing which bacteria are selected for and have the ability to persist in a host is vital when screening probiotic candidates [81]. The above prediction taxa may be good candidates for potential probiotics because they have a greater chance for colonization [17]. Therefore, targeted isolation of *Rhodobacteraceae* taxa positively selected by larval shrimp and further study on their functions and interaction with the host will help to discover novel probiotics suitable for larval shrimp nursery.

## Conclusions

To the best of our knowledge, this study is the first systematic characterization on succession patterns and assembly processes of larval shrimp bacterial community at a fine temporal scale in aquaculture practice. The diversity and composition of larval bacterial community dynamically varied with host development, with the U-shaped pattern of α-diversity, the overwhelming-dominance of *Rhodobacteraceae* since *zoea* I stage (the mouth opening of larvae), and the OTU turnover driven by temporal switching among closely related taxa as the major signatures of the succession patterns. Our results based on the neutral model revealed that the major assembly process of larval bacteria was dispersal among individuals coupled with ecological drift, while bacterioplankton also contributed to some extent during the *zoea* stage. Given the positive host selection for *Rhodobacteraceae* taxa from the rearing water during the *zoea* stage and its persistent dominance and large potential contribution to the metabolism of organic matters after the mouth opening of larvae, we suggest that *Rhodobacteraceae* could be crucial in the growth of shrimp larvae and thus be a potential source of probiotic candidates for larval nursery. Collectively, the succession patterns and assembly mechanism of larval shrimp bacteria we revealed here highlighted the importance of the mouth opening stage from the perspective of microbial ecology, indicating the possibility and timing of microbial management of the rearing water for larval microbiota regulation and pathogen prevention in larval shrimp nursery practice. One limitation of this work is that the dynamics of some important water parameters such as nutrients was not monitored corresponding to the high-frequency sampling scheme, due to the heavy workload. According to the baseline of larval bacterial community succession we revealed, future efforts should be made based on the reduced sampling frequency and more comprehensive environmental profiles for understanding the impact of water quality and feed ingredients on the succession and assembly of larval shrimp bacterial community.

## Methods

### Experimental design and sample collection

The larval shrimp nursery ponds were located in Wenchang, Hainan Province, China (20.148°N, 110.687°E). Six standardized ponds (4 m × 5 m × 1.3 m) in a larval nursery room were selected for monitoring and were maintained by the uniform management including the input and pre-treatment of seawater (before introducing larvae) and the source of eggs. This study used eggs from the same pair of parents to minimize genetic divergence and inter-individual differences. During the nursery, the rearing water was constantly aerated and maintained under the following conditions: temperature 30–32 °C, pH 8.0–8.3, salinity 30–33 PSU, and dissolved oxygen 5–8 mg/L. The concentration of inorganic nitrogen was monitored at the beginning of the nursery process, with nitrite nitrogen <0.005 mg/L and ammonia nitrogen < 0.01 mg/L. The commercial shrimp flakes were used to feed larvae at all stages after the mouth opening (6 times/day). At the stages *zoea* I-II, live microalgae *Chaetoceros* sp. and *Thalassiosira* sp. were also used (3–4 times/day). At the stages *zoea* III and *mysis*, frozen brine shrimps (*Artemia*) were used (3 times/day), and then were replaced by live brine shrimps at the early *postlarvae* stage. The initial density of larval shrimps was 3.5 million nauplii each pond with ~ 13,000 L of water. Both larvae and rearing water samples were collected across *nauplius*, *zoea* I, *zoea* II, *zoea* III, *mysis*, and early *postlarvae* stages lasting for 15 days (350 h exactly). The developmental stages of larvae were confirmed by microscopy. The strategy and timeline of the sampling are shown in Additional file 2: Figure S1 in detail. Briefly, using a dense-to-sparse sampling strategy from early to later developmental stages, 26 sampling time points were set to collect both shrimp larvae and water samples, except at the 31st, 206th, and 254th hour when only the larvae samples were collected. We randomly picked three ponds for all-time sampling, and the other three ponds were sampled at one time point of each stage (except two time points at *zoea* III, *mysis*, and early *postlarvae* stages). Shrimp larvae were soaked and washed for 10–15 s with sterilized water to remove the adsorbed rearing water, and then were transferred into sterilized and enzyme-free centrifuge tubes, followed by centrifugation at 700 rpm for 1 min, to obtain larval precipitates (about 0.8 g/pond). At the same time, rearing water samples were collected (3 L/pond) and then filtered onto a 0.2-μm polycarbonate membrane (Millipore, USA) after the pre-filtered with 100-μm sterilized nylon mesh. A total of 103 shrimp larvae samples and 92 water samples were collected and stored at – 80 °C until DNA extraction.

### DNA extraction, 16S rRNA gene amplification, and Illumina sequencing

DNA on polycarbonate membrane was extracted using the Power Soil® DNA Kit (MOBIO, USA). Total DNA of shrimp larvae was extracted using the QIAamp® DNA Stool Mini Kit (Qiagen, Germany). The V4 region of 16S rRNA genes was amplified using primers 515F-Y (5′-GTGYCAGCMGCCGCGGTAA-3′) and 806R-B (5′-GGACTACNVGGGTWTCTAAT-3′) with dual barcodes [82, 83]. To minimize reaction-level PCR bias, 10 ng purified DNA template from each sample was amplified in triplicate with a 20-μL reaction system under the following conditions: initial denaturation at 95 °C for 3 min; then 27 cycles of denaturation at 95 °C for 30 s, annealing at 55 °C for 30 s and extension at 72 °C for 45 s, with a final extension at 72 °C for 10 min. Triplicate PCR products for each sample were pooled and purified using a PCR fragment purification kit (TaKaRa, Japan). The purified PCR products were assayed for fragment size with an Agilent 2100 (Agilent, USA) and quantified using a Quant-It Pico Green kit (Invitrogen, USA) and Qubit fluorometer (Life Technologies, USA). Equimolar amounts of PCR amplicons from each sample were pooled and then sequenced on a MiSeq platform (Illumina, USA). One larvae sample at the 14th and 22nd hour and two larvae samples at the 254th hour were failed to be amplified, and thus a total of 101 larvae samples and 92 water samples were sequenced.

### Sequence processing

Raw FASTQ files were de-multiplexed with QIIME v1.9.1 [84], and the paired reads were joined using FLASH [85]. The merged sequences were quality filtered and processed with QIIME v1.9.1. Briefly, the sequences were quality-filtered using the *split_libraries_fastq.py* script at Q20 [86]. The remaining sequences were chimera detected using UCHIME [87]. After removing the chimeras, the sequences were clustered into operational taxonomic units (OTUs, 97% sequence similarity cutoff) using the *pick_open_reference_otus.py* script with the Sortmerna_sumclust method [88, 89]. The representative sequence (most abundant) of each OTU was taxonomically assigned against the SILVA 128 database [90] and aligned using PyNAST [84], respectively. A phylogenic tree was generated from the filtered alignment using FastTree [91]. Archaea, chloroplast, mitochondria, and singleton sequences were removed, as were the other sequences cannot be assigned to bacteria. The full dataset (*n* = 193) after above procedures contained 6,878,394 clean reads (mean 35,639 reads per sample). To normalize the sequencing depth, the OTU table was rarefied at 22,300 reads per sample for further analyses.

### General ecological and statistical analyses

Alpha-diversity indices (Richness, Shannon-Wiener index, phylogenetic diversity) and β-diversity metrics (Bray-Curtis dissimilarity and weighted UniFrac distance) were calculated using QIIME. Pielou’s evenness was calculated using the R package “vegan.” Kruskal-Wallis analysis was applied to test the differences in bacterial α-diversity of shrimp larvae or water among developmental stages using SPSS 22. Independent-sample *t* test was applied to test the difference in bacterial α-diversity between larvae and water at each stage. The heatmap of dominant OTUs (with average abundance > 2% at least in one sampling time point) were created using the R package “pheatmap” [92] to show the taxonomic succession of larval shrimp bacterial communities with host development, and the accessory phylogenetic tree was constructed using the maximum likelihood method in MEGA 7.0. The discriminatory taxa of each larval developmental stage were determined using linear discriminant analysis (LDA) effect size (LEfSe) [93], which employs the factorial Kruskal-Wallis sum-rank test (α = 0.05) to identify taxa with significant differences in their relative abundance at the multiple levels among the stages (using one-against-all comparisons).

Principal coordinates analysis (PCoA) based on Bray-Curtis dissimilarity and weighted UniFrac distance was applied to visualize the overall taxonomic and phylogenetic turnover of larval or water bacterial community, respectively. Analysis of similarity (ANOSIM) was performed to test the significance of differences in larval/water bacterial communities between each pair of stages or between larval and water bacterial communities at each stage using PRIMER-E v5 (PRIMER-E Ltd., UK). The time-decay model was used to evaluate the temporal turnover rate of larval bacterial community across developmental stages as following:

1 $$ S={cT}^w $$

where *S* represents the similarity of larval bacterial communities between samples over time, *T* represents the duration between observations, and the scaling exponent *w* is considered as an index of the temporal turnover rate of bacterial community and can be estimated with a linear regression based on a log-log scale:

2 $$ \log S=\log c+w\log T $$

### Inference of assembly processes of larval shrimp bacteria by the neutral model

The Sloan neutral model was used to infer the source of larval shrimp bacteria and the contribution of neutral processes (i.e., dispersal and ecological drift) to bacterial community assembly [43, 46]. For this purpose, we firstly assumed rearing water bacterioplankton as the source of larval bacterial communities. This neutral model predicts the relationship between the occurrence frequency of OTUs in larval bacterial communities and their abundance in the water bacterial communities. The goodness of model fitting was evaluated by *R*^2^, when 0 < *R*^2^ ≤ 1, the water bacteria was considered to be (one of) the important sources of larval bacterial communities. The neutral model was further used to predict the relationship between the occurrence frequency of OTUs in the local larval bacterial communities and their abundance in the wider metacommunity (the communities of all larvae sampled at a given stage). When 0 <*R*^2^ ≤ 1, the larval metacommunity was considered to be an important source of the local larval bacterial communities. In general, the model predicts that the species with high abundance in the metacommunity are more likely to disperse and be randomly sampled by hosts, while the species with low abundance are more likely to be lost from hosts due to ecological drift [46]. The R code from Burns et al. was used for neutral model fitting [46]. The 95% confidence intervals around the fitting were calculated by bootstrapping with 1000 bootstrap replicates. In the model, the estimated migration rate (*m*) was calculated using a non-linear least-squares fitting with the R package “minpack.lm” [46, 94], which is the probability that random loss of individuals in the local community will be replaced by dispersal from the metacommunity. Therefore, estimated migration rate can be interpreted as a measure of dispersal limitation, and higher *m* values means less dispersal limited [46]. In addition, we used Akaike information criterion (AIC) for comparing the goodness of fit between the binomial distribution model and the neutral model [46]. The fit of binomial distribution model presents that microbial communities were assembled only by subsampling from metacommunity (species pool) [48]. Better fit of the neutral model suggests that additional drift and dispersal limitation also contribute to local community assembly other than dispersal [46]. The OTUs that fall within the 95% confidence intervals of the best fit of the neutral model are considered as neutrally distributed. The OTUs that distribute above the 95% confidence interval (above prediction) are likely positively selected by the host or have a stronger dispersal ability relative to others. The OTUs that fall below the 95% confidence interval (below prediction) are selected against by the host or dispersal-limited from the source community.

### Functional prediction by PICRUSt2

PICRUSt2 (Phylogenetic Investigation of Communities by Reconstruction of Observed States, v2.1.0-b) pipeline was used to infer functional potentials of larval shrimp microbiota [49]. The OTU table of larval bacteria was used for predicted 16S rRNA gene copy number normalization and the functional profiles were predicted using the script *picrust2_pipeline.py*, generating a table of Kyoto Encyclopedia of Genes and Genomes (KEGG) Orthologs (KOs). Default nearest-sequenced taxon index (NSTI) options were used to filter the unreliable KO predictions. KEGG Mapper was employed to reconstruct KEGG reference categories (KEGG level 1) and modules (KEGG level 2) according to the KO annotations [95]. Subsequently, the abundances of KEGG categories and modules of bacterial community or the family *Rhodobacteraceae* were inferred by KO abundances using the custom R scripts. Then the abundances of KEGG modules of bacterial community and the contribution of the family *Rhodobacteraceae* to functional potentials were visualized using the R package “pheatmap” [92].

## Supplementary information

**Additional file 1: Table S1.** Pairwise similarity test of bacterial communities between larval shrimp developmental stages. **Table S2.** Analysis of Similarity (ANOSIM) testing the differences between larval shrimp and rearing water bacterial communities.

**Additional file 2: Figure S1.** Experimental design and sampling schedules. **Figure S2.** Dynamics of bacterial α-diversity and evenness indices of shrimp larvae and water across developmental stages. **Figure S3.** Linear discriminant analysis taxonomic cladogram showing discriminatory taxa at each developmental stage of shrimp larvae. **Figure S4.** Dynamics of bacterial community similarity among larvae samples and between larvae and water samples. **Figure S5.** The relationship between larval shrimp and water bacterial communities. **Figure S6.** Heatmaps showing the predicted function potentials of larval shrimp bacterial communities across the developmental stages.

## Data Availability

The sequence data are available in the DDBJ Sequence Read Archive under accession number DRA009910 and DRA009911 (http://ddbj.nig.ac.jp/DRASearch).
