# Supplementary material for: Fine-scale succession patterns and assembly mechanisms of bacterial community of Litopenaeus vannamei larvae across the developmental cycle
Source: Microbiome. 2020 Jul 3;8:106. doi: 10.1186/s40168-020-00879-w (PMC7334860; doi:10.1186/s40168-020-00879-w)
Supplement: Supplementary file 2 — Additional file 1: Table S1. Pairwise similarity test of bacterial communities between larval shrimp developmental stages. Table S2. Analysis of Similarity (ANOSIM) testing the differences between larval shrimp and rearing water bacterial communities. [file 40168_2020_879_MOESM1_ESM.pdf]

Additional file 1

Fine-scale succession patterns and assembly mechanisms of bacterial community of *Litopenaeus vannamei* larvae across the developmental cycle

Yanting Wang[1, 2#], Kai Wang[1, 2#*], Lei Huang[1, 2], Pengsheng Dong[1, 2], Sipeng Wang[2], Heping Chen[2, 3], Zheng Lu[4], Dandi Hou[1, 2], Demin Zhang[1, 2*]

[#]These authors contributed equally to this work.

[*]Correspondence: wangkai@nbu.edu.cn (K.W.), zhangdemin@nbu.edu.cn (D.Z.)

Supplementary Tables

Table S1 Pairwise similarity test of bacterial communities between larval shrimp developmental stages. One-way Analysis of Similarity (ANOSIM) based on Bray-Curtis dissimilarity and weighted UniFrac distance for pairwise comparison on bacterial community composition of shrimp larvae or rearing water between host developmental stages (Data present R values with ** $P < 0.01$, *** $P < 0.001$).

		Bray-Curtis dissimilarity				
	Stage	*Zoea* I	*Zoea* II	*Zoea* III	*Mysis*	*Postlarvae*
**Larvae**	*Nauplius*	0.684***	0.845***	0.939***	0.939***	0.662***
	Zoea I		0.449***	0.996***	1.000***	0.997***
	Zoea II			0.825***	0.993***	0.993***
	Zoea III				0.844***	0.986***
	Mysis					0.649***
**Water**	*Nauplius*	0.768***	0.993***	0.973***	1.000***	1.000***
	Zoea I		0.289***	0.676***	0.997***	1.000***
	Zoea II			0.538***	1.000***	1.000***
	Zoea III				0.548***	0.955***
	Mysis					0.931**
		weighted UniFrac distance				
	Stage	*Zoea* I	*Zoea* II	*Zoea* III	*Mysis*	*Postlarvae*
**Larvae**	*Nauplius*	0.493***	0.729***	0.768***	0.536***	0.342***
	Zoea I		0.196***	0.344***	0.389***	0.592***
	Zoea II			0.319***	0.521***	0.763***
	Zoea III				0.516***	0.802***
	Mysis					0.382***
**Water**	*Nauplius*	0.822***	0.994***	0.784***	0.998***	1.000***
	Zoea I		0.147***	0.629***	0.910***	0.934***
	Zoea II			0.704***	1.000***	1.000***
	Zoea III				0.520***	0.780***
	Mysis					0.470**

Table S2 Analysis of Similarity (ANOSIM) testing the differences between larval shrimp and rearing water bacterial communities.

	Bray-Curtis dissimilarity		weighted UniFrac distance	
	R	*P*	R	*P*
Nauplius	0.713	0.001	0.619	0.001
Zoea I	0.753	0.001	0.615	0.001
Zoea II	0.933	0.001	0.803	0.001
Zoea III	0.935	0.001	0.579	0.001
Mysis	1.000	0.002	0.993	0.001
Postlarvae	0.967	0.001	0.834	0.001
