# Supplementary material for: Fine-scale succession patterns and assembly mechanisms of bacterial community of Litopenaeus vannamei larvae across the developmental cycle
Source: Microbiome. 2020 Jul 3;8:106. doi: 10.1186/s40168-020-00879-w (PMC7334860; doi:10.1186/s40168-020-00879-w)
Supplement: Supplementary file 3 — Additional file 2: Figure S1. Experimental design and sampling schedules. Figure S2. Dynamics of bacterial α-diversity and evenness indices of shrimp larvae and water across developmental stages. Figure S3. Linear discriminant analysis taxonomic cladogram showing discriminatory taxa at each developmental stage of shrimp larvae. Figure S4. Dynamics of bacterial community similarity among larvae samples and between larvae and water samples. Figure S5. The relationship between larval shrimp and water bacterial communities. Figure S6. Heatmaps showing the predicted function potentials of larval shrimp bacterial communities across the developmental stages. [file 40168_2020_879_MOESM2_ESM.pdf]

Additional file 2

Fine-scale succession patterns and assembly mechanisms of bacterial community of *Litopenaeus vannamei* larvae across the developmental cycle

Yanting Wang[1, 2#], Kai Wang[1, 2#*], Lei Huang[1, 2], Pengsheng Dong[1, 2], Sipeng Wang[2], Heping Chen[2, 3], Zheng Lu[4], Dandi Hou[1, 2], Demin Zhang[1, 2*]

[#]These authors contributed equally to this work.

[*]Correspondence: wangkai@nbu.edu.cn (K.W.), zhangdemin@nbu.edu.cn (D.Z.)

Supplementary Figures

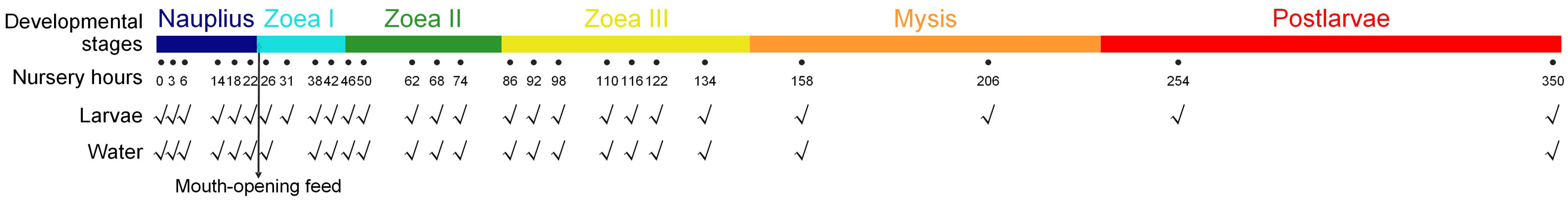

Figure S1 Experimental design and sampling schedules. The "Larvae" and "Water" rows show the time points when shrimp larvae and/or water samples were collected.

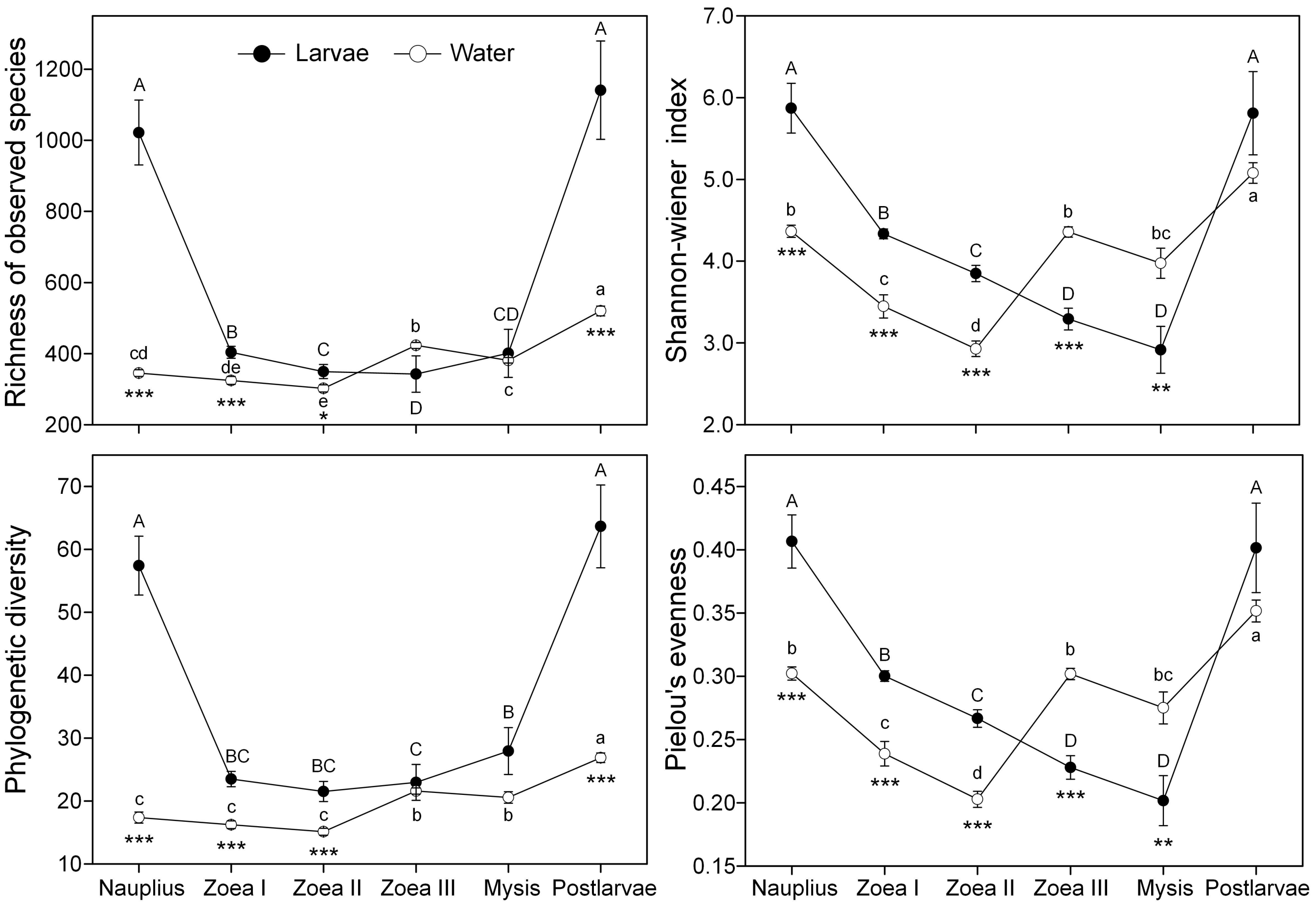

Figure S2 Dynamics of bacterial α-diversity and evenness indices of shrimp larvae and water across developmental stages. Data present means ± standard errors. The different letters indicate significant difference between stages ($P < 0.05$) (uppercase for larvae; lowercase for water samples). Significance of differences between larvae and water at each stage was tested using Independent-Sample *t*-test (* $P < 0.05$, ** $P < 0.01$, *** $P < 0.001$).

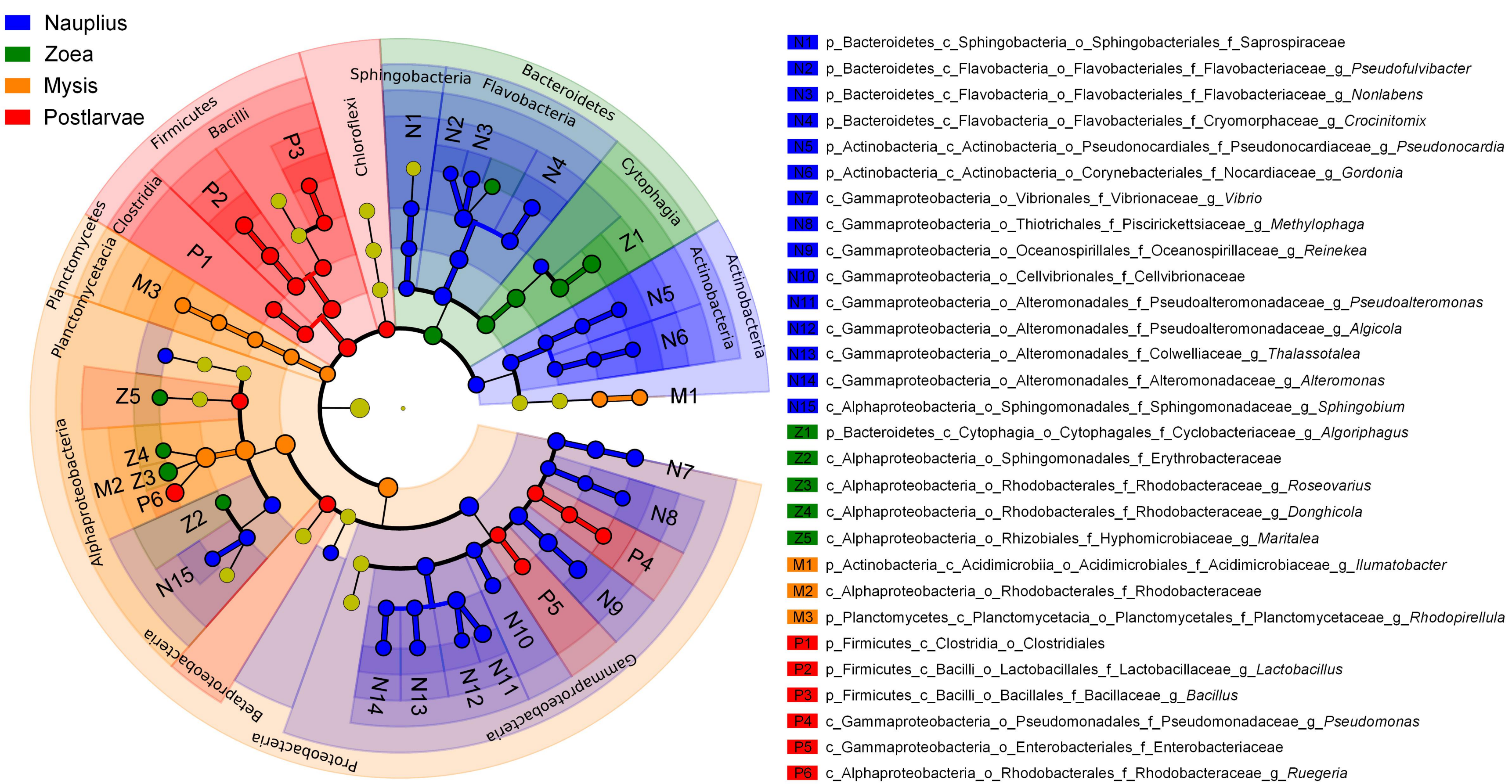

Figure S3 Linear discriminant analysis taxonomic cladogram showing discriminatory taxa at each developmental stage of shrimp larvae. The taxon with average abundance > 2% at least in one sampling time point are shown. Discriminatory taxon nodes are coloured and branch areas are shaded according to the highest ranked group for that taxon. If the taxon is not significantly differentially represented among sample groups, the corresponding node is coloured yellow.

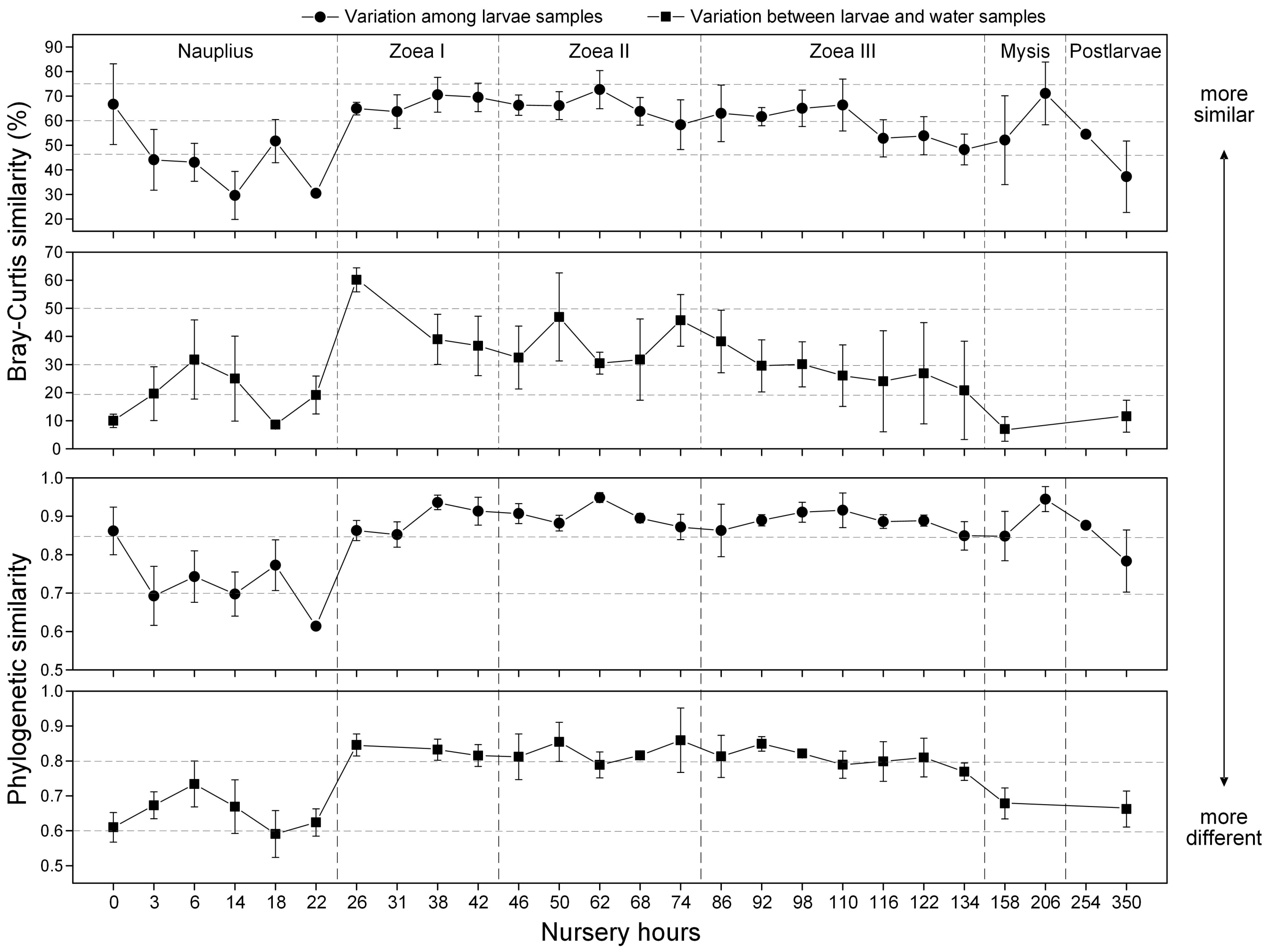

Figure S4 Dynamics of bacterial community similarity among larvae samples and between larvae and water samples. Bray-Curtis similarity and phylogenetic similarity based on UniFrac distance (1 - weighted UniFrac distance) are shown. Data present means ± standard deviation.

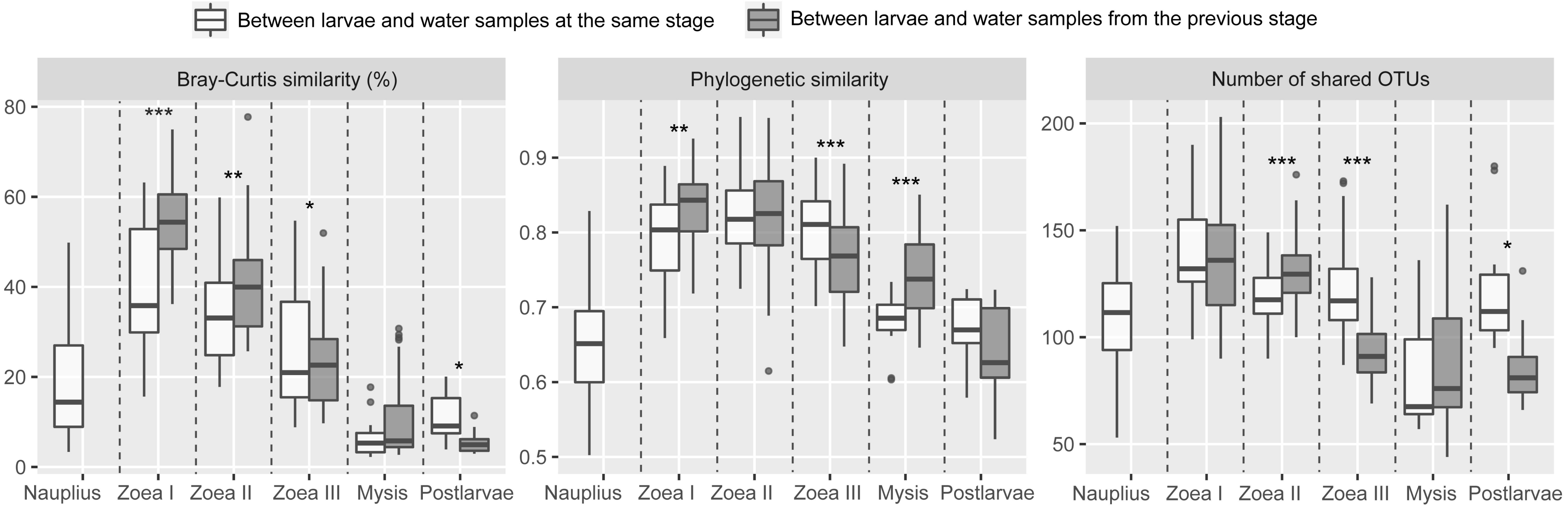

Figure S5 The relationship between larval shrimp and water bacterial communities. Box plots showing Bray-Curtis similarity, phylogenetic similarity based on UniFrac distance (1 - weighted UniFrac distance), and number of shared OTUs of shrimp larvae with water samples at the same stage or from the previous stage. Statistical significance was tested by Independent-Sample *t*-test (* $P < 0.05$, ** $P < 0.01$, *** $P < 0.001$). The lower and upper hinges correspond to the first and third quartiles (the 25th and 75th percentiles). The upper whisker extends from the hinge to the largest value no further than 1.5 × IQR (inter-quartile range) from the hinge. The lower whisker extends from the hinge to the smallest value at most 1.5 × IQR of the hinge. Data beyond the end of the whiskers are 'outlying' points and are plotted individually.

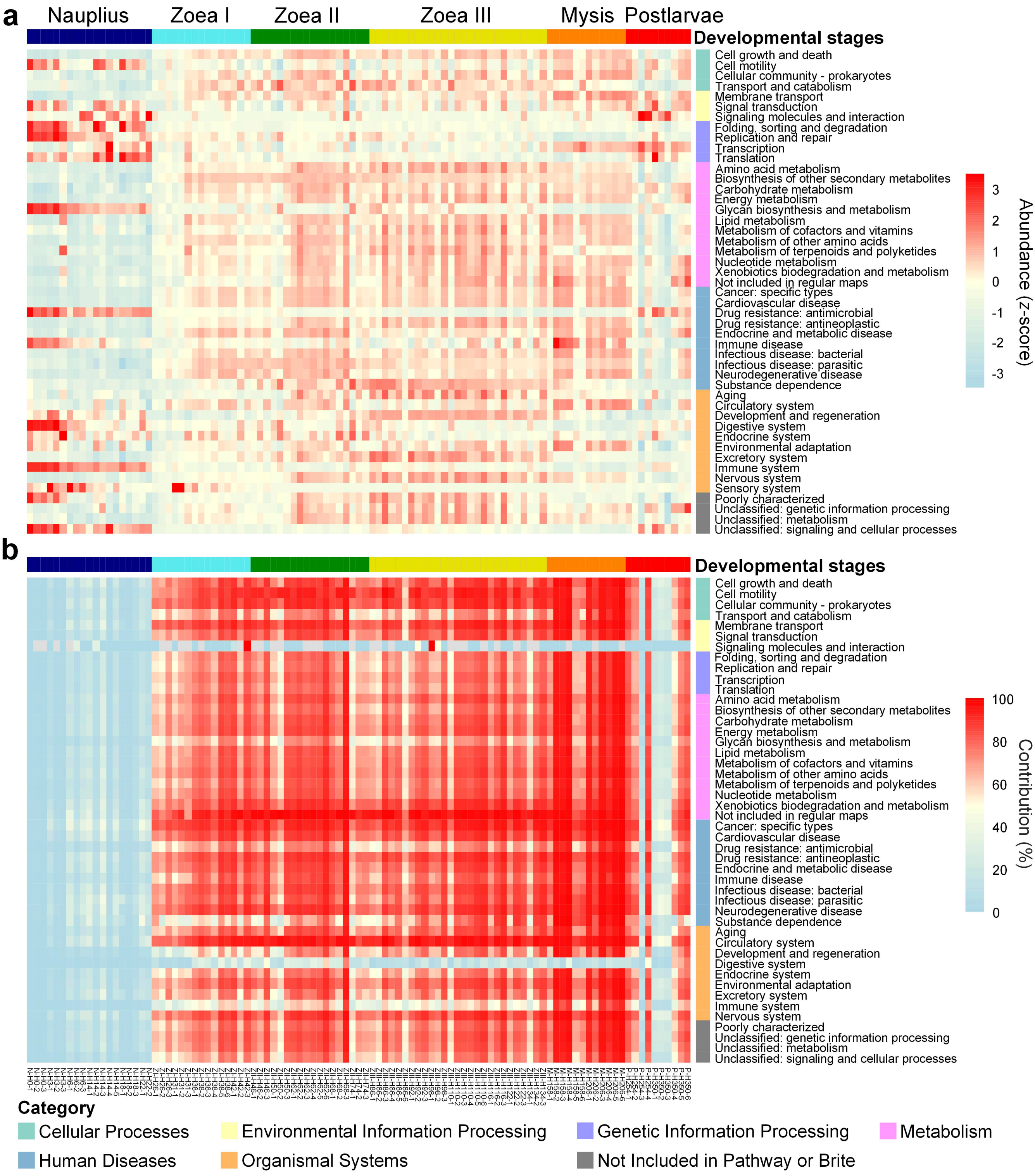

Figure S6. Heatmaps showing the predicted function potentials of larval shrimp bacterial communities across the developmental stages. **a** The predicted abundance (*z*-score transformed) of KEGG modules (level 2) of the whole bacterial community of larval shrimps. **b** The contribution of the family *Rhodobacteraceae* to the functional potentials of bacterial community.
